# Canadian Assessment of Physical Literacy Second Edition: a streamlined assessment of the capacity for physical activity among children 8 to 12 years of age

**DOI:** 10.1186/s12889-018-5902-y

**Published:** 2018-10-02

**Authors:** Patricia E. Longmuir, Katie E. Gunnell, Joel D. Barnes, Kevin Belanger, Geneviève Leduc, Sarah J. Woodruff, Mark S. Tremblay

**Affiliations:** 1 Healthy Active Living and Obesity Research Group, Children’s Hospital of Eastern Ontario Research Institute, 401 Smyth Road, Ottawa, ON K1H 8L1 Canada; 2 Department of Paediatrics, Faculty of Medicine, University of Ottawa, Ottawa, ON K1H 8M5 Canada; 3 Department of Psychology, Carleton University, Ottawa, ON K1S 5B6 Canada; 4 Department of Kinesiology, University of Windsor, Windsor, ON N9B 3P4 Canada

**Keywords:** Physical activity, Physical competence, Daily behaviour, Motivation and confidence, Knowledge and understanding

## Abstract

**Background:**

The Canadian Assessment of Physical Literacy (CAPL) assesses the capacity of children to lead a physically active lifestyle. It is comprised of a battery of standardized assessment protocols that reflect the Canadian consensus definition of physical literacy. The Royal Bank of Canada Learn to Play - Canadian Assessment of Physical Literacy study implemented the CAPL with 10,034 Canadian children (50.1% female), 8 to 12 years of age. Feedback during data collection, necessary changes identified by the coordinating centre, and recent data analyses suggested that a streamlined, second edition of the CAPL was required. The purpose of this paper is to describe the methods used to develop the CAPL second edition (CAPL-2).

**Methods:**

The larger dataset created through the RBC–Learn to Play CAPL study enabled the re-examination of the CAPL model through factor analyses specific to Canadian children 8 to 12 years of age from across Canada. This comprehensive database was also used to examine the CAPL protocols for redundancy or variables that did not contribute significantly to the overall assessment. Removing redundancy had been identified as a priority in order to reduce the high examiner and participant burden. The “lessons learned” from such a large national surveillance project were reviewed for additional information regarding the changes that would be required to optimize the assessment of children’s physical literacy. In addition, administrative changes, improvements, and corrections were identified as necessary to improve the quality and accuracy of the CAPL manual and training materials.

**Results:**

For each domain of the CAPL, recommended changes based on the factor analyses, qualitative feedback and theoretical considerations significantly reduced the number of protocols. Specific protocol combinations were then evaluated for model fit within the overarching concept of physical literacy. The CAPL-2 continues to reflect the four components of the Canadian consensus definition of physical literacy: Motivation and Confidence, Physical Competence, Knowledge and Understanding, and engagement in Physical Activity Behaviour. The CAPL-2 is comprised of three Physical Competence protocols (plank, Progressive Aerobic Cardiovascular Endurance Run [PACER], Canadian Agility and Movement Skill Assessment [CAMSA]), two Daily Behaviour protocol (pedometer steps, self-reported physical activity), and a 22-item questionnaire assessing the physical literacy domains of Motivation and Confidence, and Knowledge and Understanding. Detailed information about the CAPL-2 is available online (www.capl-eclp.ca).

**Conclusions:**

The CAPL-2 dramatically reduces examiner and participant burden (three Physical Competence protocols, two Daily Behaviour protocols, and a 22-response questionnaire; versus eight Physical Competence protocols, three Daily Behaviour protocols and a 72-response questionnaire for the original CAPL), while continuing to be a comprehensive assessment of all aspects of children’s physical literacy using the Canadian consensus definition of this term. Like the original, the CAPL-2 continues to offer maximum flexibility to practitioners, who can choose to complete the entire CAPL-2 assessment, only one or more domains, or select individual protocols. Regardless of the assessment selected, scores are available to interpret the performance of each child relative to Canadian children of the same age and sex. All of the protocols included in the CAPL-2 have published reports of validity and reliability for this age group (8 to 12 years). The detailed manual for CAPL-2 administration, along with training materials and other resources, are available free of charge on the CAPL-2 website (www.capl-eclp.ca). All CAPL-2 materials and resources, including the website, are available in both English and French.

**Electronic supplementary material:**

The online version of this article (10.1186/s12889-018-5902-y) contains supplementary material, which is available to authorized users.

## Background

The Canadian Assessment of Physical Literacy (CAPL) assesses the capacity of children to lead a physically active lifestyle [1]. Canadian organizations have achieved consensus that physical literacy should be defined as the “motivation, confidence, physical competence, knowledge and understanding to value and take responsibility for engagement in physical activities for life” [2]. This definition is adopted from the International Physical Literacy Association [3]. The CAPL is comprised of a battery of standardized assessment protocols that have published validity and reliability for children 8 to 12 years of age [4], and reflect this internationally accepted definition of physical literacy. Since 2013, the CAPL has been used to assess the physical literacy of more than 10,000 children in Canada and abroad (e.g., Australia, Kenya, South Africa, United Kingdom, Singapore) [5].

## Methods

The Royal Bank of Canada Learn to Play–Canadian Assessment of Physical Literacy (RBC Learn to Play–CAPL) project was designed to survey the capacity of Canadian children to engage in physical activity, sport, and recreation opportunities. With support from RBC, the Public Health Agency of Canada, ParticipACTION, and Mitacs, research teams led by Site Principal Investigators from 11 post-secondary institutions in seven Canadian provinces assessed the physical literacy of children aged 8 to 12 years using the CAPL. Children within this sample were primarily recruited through elementary schools; however, summer camps and after-school programs were also targeted.

During the four years of data collection for the RBC Learn to Play–CAPL project, Site Principal Investigators relayed feedback on administration of the CAPL to the coordinating centre (i.e., the Healthy Active Living and Obesity Research Group in Ottawa, Ontario). In light of these “lessons learned” [5], as well as desired changes identified by the coordinating centre and recent data analyses (confirmatory factor analysis and measures of model fit [6, 7]), a second edition of the CAPL (CAPL-2) was developed. The aim of this paper is to describe the CAPL-2, which replaces the original CAPL, and can be used as a valid and reliable assessment of physical literacy in children.

### Rationale for revising the Canadian assessment of physical literacy

When the CAPL was originally developed, item scoring for the protocols was based on results from a factor analysis of 489 Canadian children (8 to 12 years of age; 58% female) assessed in the Ottawa area [4]. However, limitations of the original item scoring protocols were apparent given that the children had been recruited from only one region of the country, and there were limited numbers of males and females across the full age spectrum (8 to 12 years). At the time, a Delphi process recommended that additional analyses be performed in the future to evaluate the model fit of the CAPL and the appropriateness of the established scoring system [8]. The availability of a much larger dataset, collected from sites across Canada during the RBC Learn to Play – CAPL, provided the opportunity to re-examine model fit through factor analyses [6] and to utilize data specifically from children 8 to 12 years of age in order to establish a scoring system based on normative data.

The availability of such a comprehensive database also provided the opportunity to examine the CAPL protocols for redundancy or variables that did not contribute significantly to the overall assessment. Removing redundancy within the CAPL was seen as a critical next step given that a major critique of the CAPL had been the high examiner and participant burden [9]. Although the CAPL was designed for flexibility and modular implementation (i.e., using any of the protocols or domains, either singly or in combination, or the full assessment), there was particular concern about the time required to complete all protocols, the skill/training needed to conduct the Canadian Agility and Movement Skill Assessment (CAMSA), the requirement for two examiners to conduct the CAMSA, and prohibitions for measuring height and weight in some settings (e.g., schools). We therefore sought to refine the CAPL through balancing the recommendations made by experts through a Delphi process to include objective measures of all major domains [8] within the International Physical Literacy Association [3] and Canadian Consensus definition of physical literacy [2], and the criticism about high examiner and participant burden conveyed from users of the CAPL. In examining the experts’ advice, users’ experience, and physical literacy research, we recognized that the body composition protocols initially recommended through the Delphi process (body mass index and waist circumference) stemmed from a health assessment framework and, as such, were more peripheral than direct indicators of physical literacy [4]. Similarly, the “lessons learned” from completing such a large national surveillance project [5] provided additional information on changes that could optimize the measurement of children’s physical literacy in the future (see Results section).

Finally, there were administrative changes, improvements, and corrections that had been identified as necessary to improve the quality and accuracy of the CAPL manual and training materials, which are publicly available. Notably, the name for the obstacle course was updated to become the CAMSA, and agreement was reached that the French translation of “physical literacy” would be “littératie physique” (formerly “savoir-faire physique”). Additionally, two errors identified in the scoring and documentation for the CAPL questionnaire also required correction. One item, which asked children about the activities they would do after school if they could choose from a list of active and sedentary pursuits, had been included in the Knowledge and Understanding domain of the original CAPL even though it was intended to be part of the Motivation and Confidence domain [8]. The second error was a statement in the CAPL manual that the barriers instrument should be reverse scored. The CAPL website scored the instrument correctly but the instructions in the manual were incorrect for those wishing to do the scoring manually. Subsequently, the objective set forth by the creators of the CAPL was to relaunch a leaner, more efficient tool that reduced administrator and participant burden while maintaining alignment with the internationally accepted definition of physical literacy.

## Results

### Canadian Assessment of Physical Literacy, Second Edition (CAPL-2)

The CAPL-2 continues to be anchored in the Canadian consensus definition of physical literacy [2] which matches that developed by the International Physical Literacy Association [3]. That is, the four domains of the CAPL-2 reflect the child’s i) motivation and confidence, ii) physical competence, and iii) knowledge and understanding, to take responsibility for iv) engagement in physical activities for life [2]. The CAPL-2 is comprised of three Physical Competence protocols, two Daily Behaviour protocols, and a questionnaire requiring responses to 22 items assessing knowledge and understanding and motivation and confidence. In contrast, the original CAPL included eight Physical Competence protocols, three Daily Behaviour protocols, and a questionnaire requiring 72 responses. The protocols included in the CAPL-2 are described below. Detailed information about the CAPL-2 is available online (www.capl-eclp.ca). The theoretical and statistical rationales for these changes are provided in detail by Gunnell et al. [6]. In brief, confirmatory factor analysis provided validity evidence for a shorter, more concise CAPL. The analysis found that measures of flexibility, grip strength, and safety gear used during physical activity did not contribute significantly to the domain or overall CAPL scores. As well, while items assessing body composition, knowledge of definitions of health, and self-reported sedentary behaviour were aligned with the assessment of health outcomes, they were not theoretically linked to the concept of physical literacy.

#### Motivation and Confidence

The Motivation and Confidence domain of CAPL-2 is assessed by 12 items within the CAPL-2 self-report questionnaire (see pages 3 and 4 of Additional file 1). Four aspects of motivation and confidence are evaluated, with each being assessed via three items. *Predilection* assesses the child’s preference for physically active pursuits. *Adequacy* assesses their expectations for success. *Perceived competence satisfaction* assesses whether children perceive they can complete optimally challenging physical activities. *Intrinsic motivation* assesses the degree to which children pursue activity for its own sake (i.e., for fun or enjoyment) rather than for some other outcome (e.g., pressure from parents). The items included in the Motivation and Confidence domain have been extracted from previously published instruments [10, 11], and have been modified based on recent data analyses completed as part of the RBC Learn to Play–CAPL project [7].

Predilection and adequacy are assessed using a structured alternative-response format (see page 3 of Additional file 1). For each item, children are presented with two descriptions of what “some children” enjoy or do, and they are asked to choose the children that are most similar to themselves. For example, “Some kids do well in most sports but other kids feel they aren’t good at sports”. After choosing which children are most similar to themselves, children are asked to indicate whether the statement is either “really true” or “sort of true” for them.

Perceived competence and intrinsic motivation are assessed using a 5-point Likert-type scale, with response options ranging from “Not like me at all” to “Really like me” (see page 4 of Additional file 1). Children are provided with a series of positively framed statements, and asked to rate each statement as to whether it is or is not similar to themselves. For example, “When it comes to being active, I have good skills”.

**Fig. 1:**
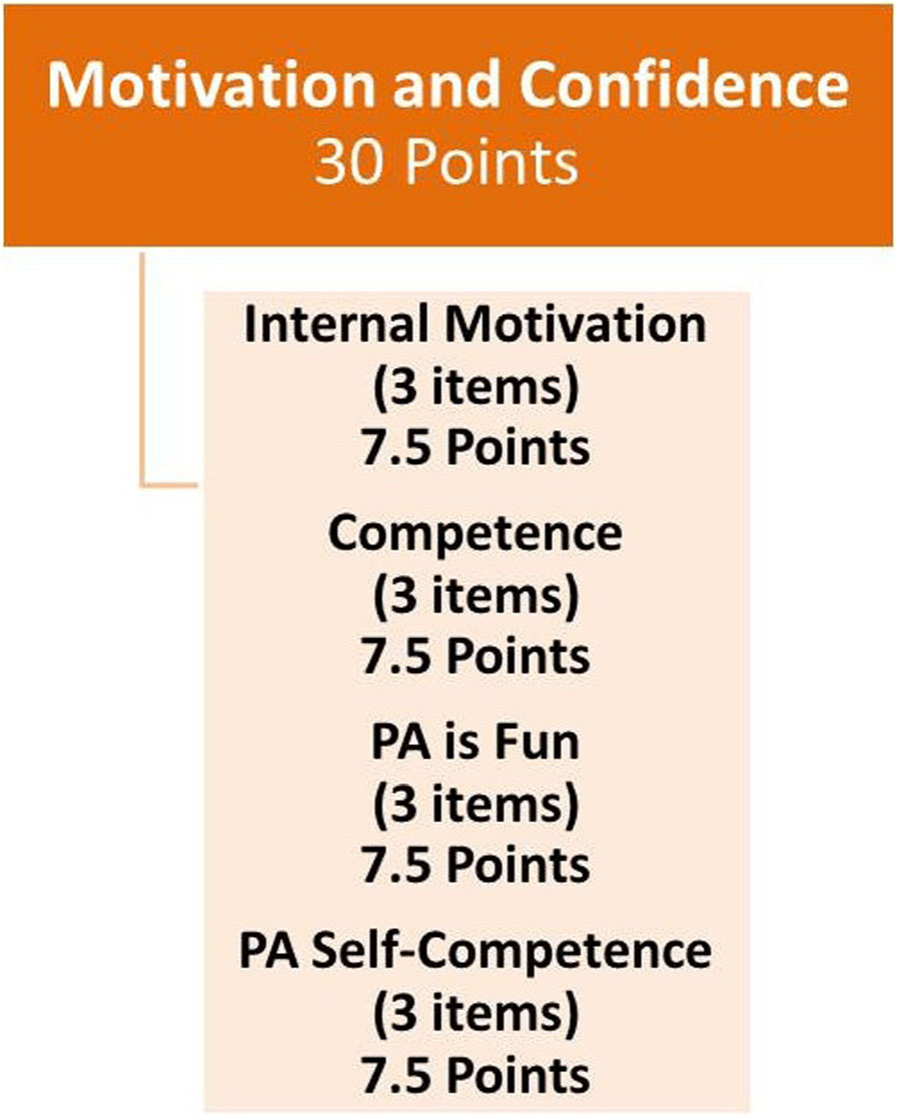
Motivation and Confidence scoring. PA: physical activity

Each of the 12 items within the Motivation and Confidence assessment is assigned a maximum of 2.5 points, such that the maximum total score for the domain is 30 points (see Fig. 1). For predilection (Physical Activity [PA] is Fun) and adequacy (PA Self-Competence) items, the points awarded for each response are 0.6, 1.2, 1.8 or 2.5 points, respectively, with responses indicating a preference or positive view of physical activity being awarded more points. For perceived competence and intrinsic motivation, the 5-point Likert scale is assigned 0.5 to 2.5 points, with higher scores on the Likert-scale representing higher motivation and competence.

#### Physical Competence

**Fig. 2:**
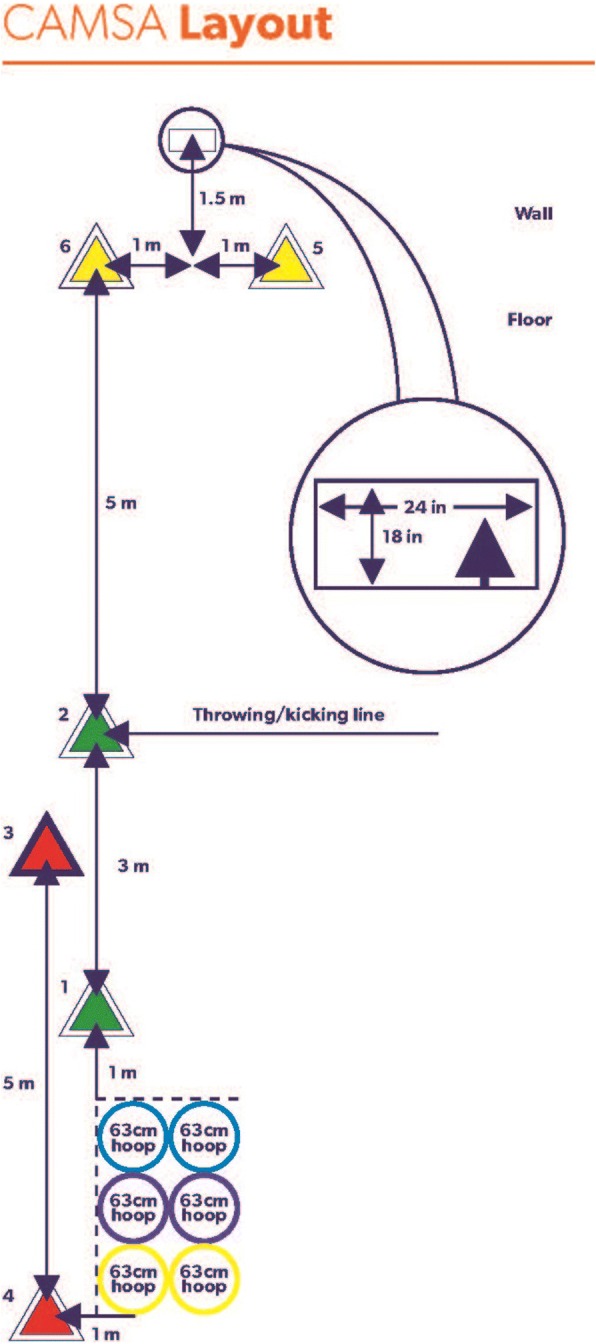
Canadian Agility and Movement Skill Assessment. Permission to reprint this figure has been provided by Mark Tremblay and Patricia Longmuir. Reprinted from the Canadian Assessment of Physical Literacy Manual for Test Administration, second edition. Healthy Active Living and Obesity Research Group. 2017. https://www.capl-eclp.ca/wp-content/uploads/2017/10/capl-2-manual-en.pdf. Accessed 15 Jan 2018

Physical Competence is assessed through the completion of three protocols. Fundamental, complex and combined movement skills are assessed with the CAMSA (Fig. 2) [12]. The Progressive Aerobic Cardiovascular Endurance Run (PACER) protocol assesses aerobic endurance [13]. The isometric plank hold assesses muscular endurance [14]. These protocols were all included in the original version of the CAPL, and the administration of these protocols has not changed for the CAPL-2. New to CAPL-2 is the revised scoring system structure, whereby each protocol in the Physical Competence assessment is now assigned a score out of 10 points, for a maximum total score for the domain of 30 points. Points awarded for each protocol are based on the range of values observed among the more than 10,000 Canadian children assessed to date. For example, if the child holds the plank position for less than 20 s, 0 points are awarded. A 60-s plank hold is awarded 5 points, and a hold of over 110 s is required to achieve the maximum score of 10 points. Brief descriptions of each protocol are provided here, with detailed instructions available on the CAPL website (www.capl-eclp.ca), in the CAPL training videos and manual.

##### Canadian Agility and Movement Skill Assessment

To complete the CAMSA assessment of movement skill, children are asked to perform a sequence of physical activity skills. Fundamental movement skills include jumping on two feet (three hoops on right side), sliding sideways (between green cones), catching, overhand throw, skipping (between red cones), hopping on one foot (all hoops), and kicking a ball. Within the setting of the agility course, children must also perform more complex movement skills, such as acceleration, deceleration, dynamic balance, and transitions. The quality of each of the fundamental movement skills performed is scored based on 14 specific movement criteria (each criterion receives 1 point if performed correctly). The time required to complete the agility course (from “Go” command to contact with ball for the kick) is recorded as an indication of performance of the complex movement skills. Children with lower levels of physical literacy will either move quickly and perform the skills poorly, or go very slowly in order to perform the skills correctly. Children with higher levels of physical literacy are better able to select the optimal speed that will maximize both skill performance and completion time. Children are given two practice trials before completing two timed and scored trials. The examiner prompts each skill as it is to be performed during each trial, to ensure that performance is influenced by movement skill rather than memory.

##### Isometric plank hold

**Fig. 3:**
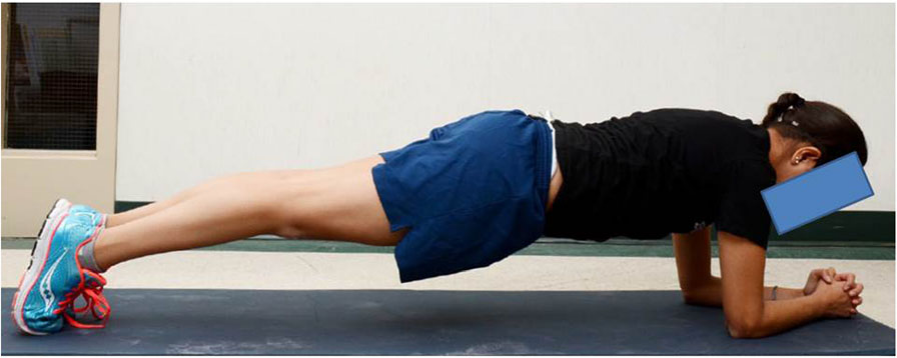
Isometric plank hold

The plank isometric hold is a timed assessment of the maximum time that the child can maintain the correct body position. The body is held in a straight line from ears to ankles, supported only on the forearms and toes (Fig. 3). Children are given one short practice to allow them to learn the correct body position. The timing starts when they are in the correct position, and continues until test termination. The first time the child’s body position becomes incorrect (e.g., hips are too high or too low; legs bent), the examiner prompts the child to correct the position. The second time the position is incorrect, the test is terminated.

##### PACER

**Fig. 4:**
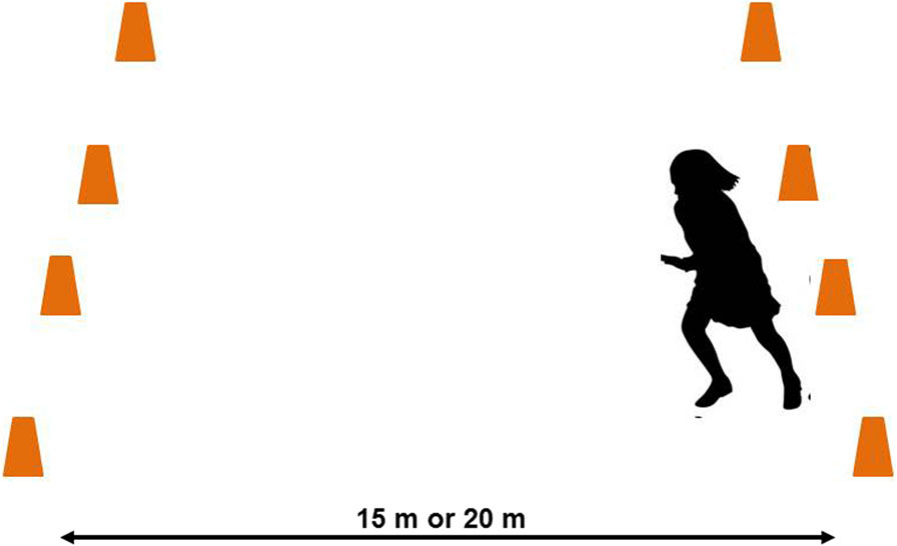
Progressive Aerobic Cardiovascular Endurance Run

The PACER requires the child to run back and forth across an open space at a speed designated by the test protocol (Fig. 4). The distance across the open space can be either 15 m or 20 m, with the speed signal and scoring adjusted according. Children must have their foot across the line on the opposite side of the open space before the next signal to reverse direction. The first time the child does not reach the line before the signal, the child is prompted to reverse direction and run more quickly. The second time the child does not reach the line before the signal, the test is terminated. The number of lengths completed, including the first length that did not reach the line, is recorded.

#### Knowledge and Understanding

**Fig. 5:**
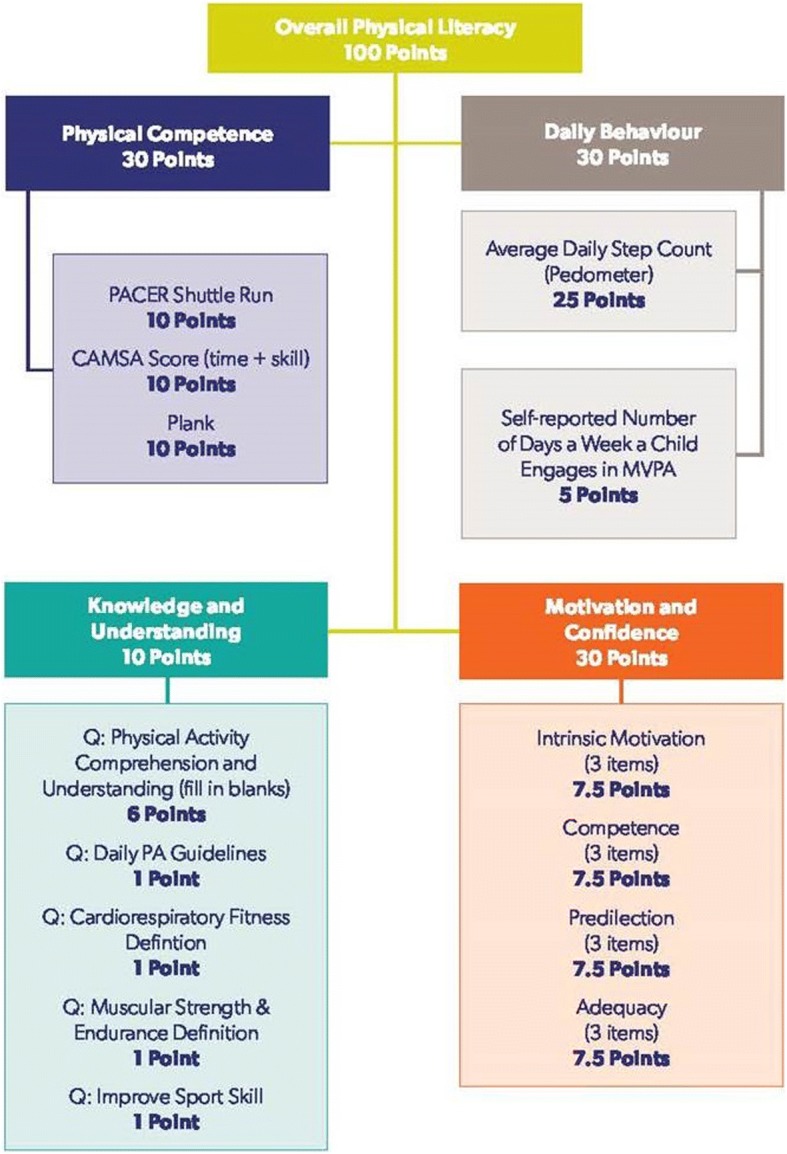
CAPL-2 scoring. CAMSA: Canadian Agility and Movement Skill Assessment; MVPA: Moderate-to-vigorous physical activity; PACER: Progressive Aerobic Cardiovascular Endurance Run

The assessment of the child’s knowledge and understanding of physical literacy (pages 5 and 6 in Additional file 1) continues to reflect the content of Canadian physical education curricula [15] as well as the recommendations of an international Delphi panel [8]. For CAPL-2, the knowledge assessment focuses on four areas: recommended daily physical activity; terminology related to aerobic endurance; terminology related to muscular endurance; and methods to enhance physical competence. In order to focus more explicitly on knowledge and understanding of physical activity constructs, and based on theoretical considerations and the results of an updated factor analysis, questions about health in general, safety during physical activity, and screen time were removed [6]. Four of five questions are assessed using a multiple-choice question format. The response options for the question assessing knowledge of the physical activity guidelines were changed to ensure that the correct answer was not the highest number of minutes. Asking children to fill in the missing words to create a short paragraph continues to evaluate their knowledge of how to enhance specific aspects of physical competence, with one additional response required for CAPL-2. Details of the changes to the Knowledge and Understanding assessment have been published by Longmuir et al. [15]. Each correct response in the Knowledge and Understanding assessment is assigned 1 point, for a maximum score of 10 points (see Fig. 5).

#### Daily Behaviour

Assessments of sedentary behaviour have been removed from the Daily Behaviour domain, as additional evidence has emerged that sedentary behaviour and physical activity are not opposite ends of the same continuum [16], but rather independent variables affecting lifestyle and future health. Daily physical activity behaviour is objectively assessed from pedometer step counts. Children are asked to wear a pedometer during all waking hours for seven consecutive days (five days at school and two weekend days). Pedometer data are included in the analysis if the child wore the pedometer for at least three days with a minimum of 10 h of pedometer wear per day, and the recorded number of steps is between 1000 and 30,000 [17]. Pedometers are the measure of choice for the CAPL because they are inexpensive and can easily be implemented by teachers or physical activity leaders. However, other devices (such as accelerometers) may also be used if available in order to determine whether children meet the recommended level of daily physical activity. One item in the CAPL-2 questionnaire is also used to assess physical activity behaviour. As recommended by the international Delphi panel [8], children are asked to self-report the number of days in the past week that they were physically active for at least 60 min per day. The child’s average daily step count from the pedometer assessment is awarded a maximum of 25 points (from 0 points for < 2000 steps to 25 points for > 17,999 steps), with higher points assigned for the performance of more steps per day and 17 points awarded for achieving the recommended 12,000 steps per day. The self-report of physical activity is assigned a maximum of 5 points, for a total maximum score for the Daily Behaviour domain of 30 points (see Fig. 5).

#### CAPL-2 scoring

One marked improvement of the CAPL-2 is that the scoring system is now informed by data for each CAPL protocol as completed by more than 10,000 Canadian children. As recommended by the Delphi panel [8], children perform the same assessment activities at all ages, but the scoring, interpretation, and feedback provided varies by age and sex. Performance standards for daily pedometer step counts were based on research recommendations [18]. Until research data are available linking the other physical literacy measures within CAPL-2 to criterion or desired outcomes, we have defined the interpretive categories for all other protocols based on the normative CAPL data collected to date from Canadian children. This has enabled the provision of age- and sex-specific scoring percentiles based on generalized additive models for location, scale, and shape (GAMLSS). The scores for each protocol, domain scores, and the overall CAPL-2 score continue to be interpreted within four categories as follows:

- Beginning = less than the 17th percentile.
- Progressing = 17th to 65th percentiles.
- Achieving = above the 65th percentile to the 85th percentile.
- Excelling = above the 85th percentile.

**Fig. 6:**
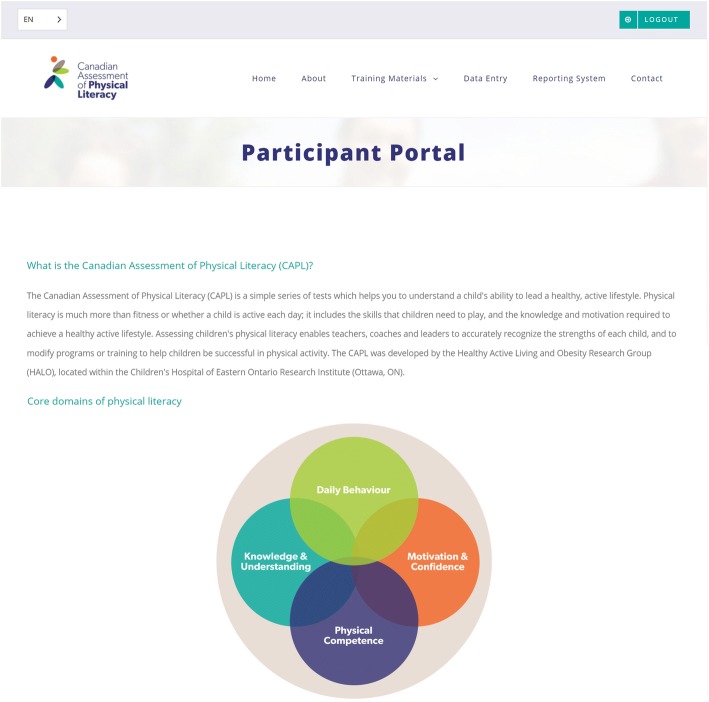
Individual report of CAPL-2 results – participant portal

**Fig. 7:**
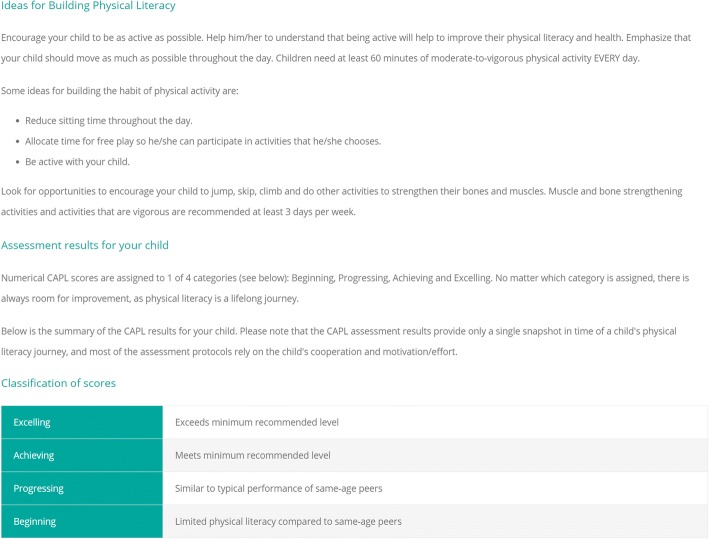
Individual report of CAPL-2 results – ideas for building physical literacy

**Fig. 8:**
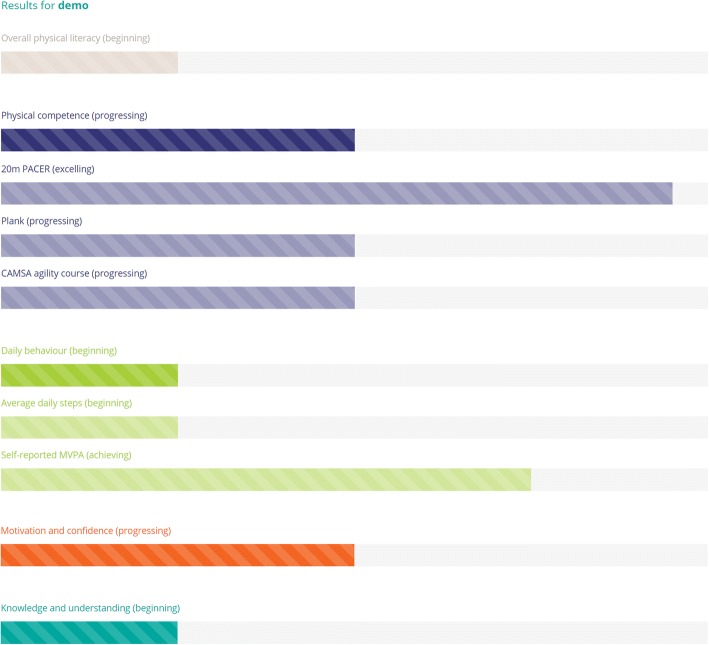
Individual report of CAPL-2 results – example of individual results. CAMSA: Canadian Agility and Movement Skill Assessment; MVPA: moderate to vigorous physical activity; PACER: Progressive Aerobic Cardiovascular Endurance Run

**Fig. 9:**
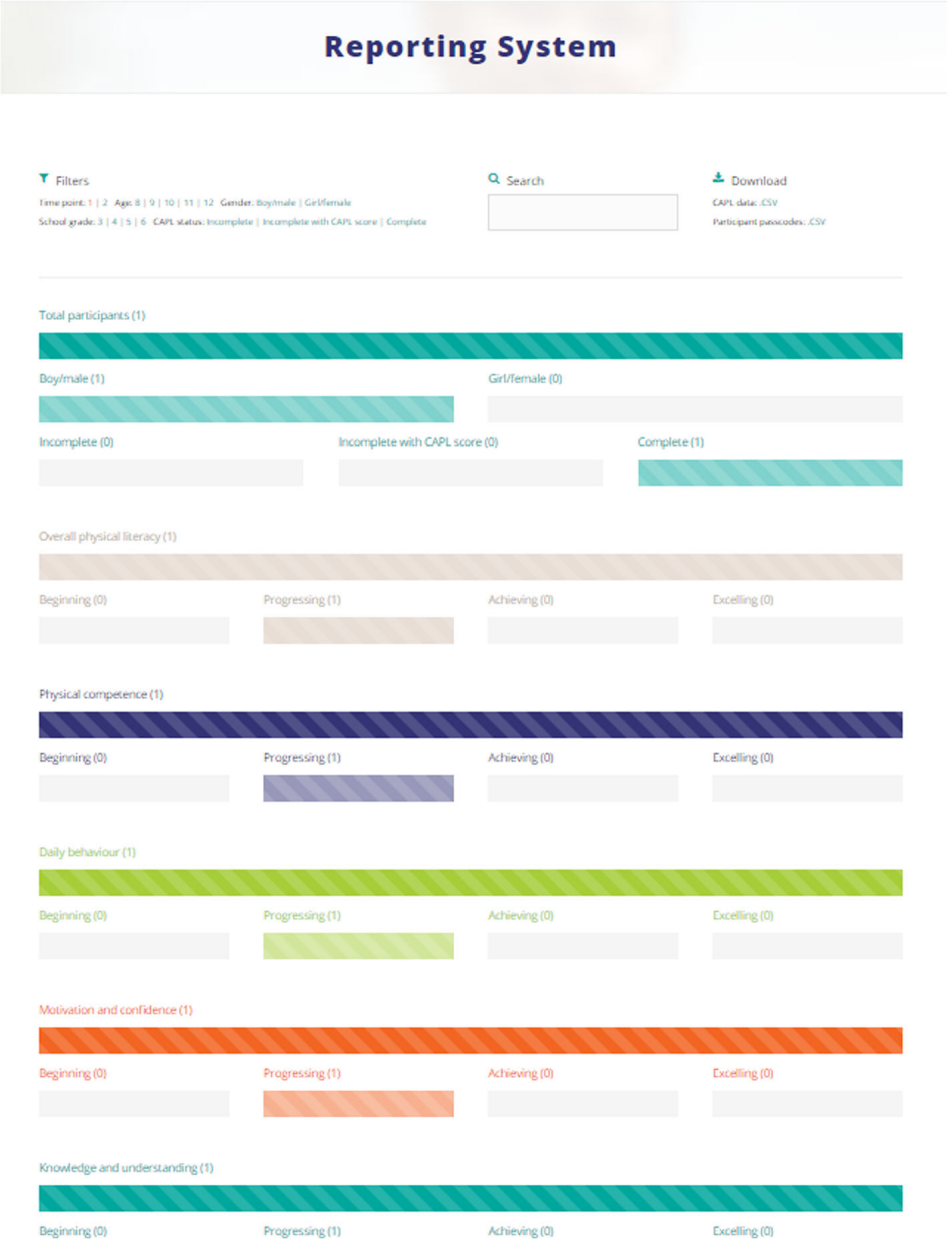
CAPL-2 Example of summary of results for a group of participants

Once participant data are collected, CAPL scores can be automatically calculated by entering the data into the online CAPL-2 website (www.capl-eclp.ca), which will also generate a summary of assessment results for individual children (Figs. 6, 7, and 8) or groups of children (Fig. 9). This revised scoring system has also been implemented for all of the protocols in the original CAPL (see www.capl-ecsfp.ca), for those still wishing to use the original assessment tool.

#### CAPL-2 training and support materials

All of the training and support materials required for implementation of the CAPL-2 are available free of charge on the CAPL-2 website (www.capl-eclp.ca). The materials available include the detailed manual for CAPL-2 administration, a quick-start guide for the CAPL-2 protocols, and training videos for each CAPL-2 protocol (plank, PACER, CAMSA, pedometer, questionnaire). All of these materials have been updated to reflect the changes implemented for the CAPL-2, and all are available in both English and French.

### Study strengths and limitations

To our knowledge, the CAPL (editions 1 and 2) continues to be one of the only physical literacy assessments based solely on protocols with peer-reviewed evidence of validity and reliability. Furthermore, both editions have undergone extensive scientific examination and cross-validation across numerous samples, regions of Canada, and iterations of the assessment protocol [6–8, 12, 14, 15]. A key strength of the CAPL-2 is that the selection of protocols for inclusion, as well as scoring and performance standards, were based on a large database of more than 10,000 children who were assessed at 11 sites across Canada through the RBC Learn to Play–CAPL project.

A limitation of the RBC Learn to Play–CAPL project is that participants were recruited from convenience samples of children attending local schools, camps, or child care programs, so the extent to which the sample is representative of the Canadian population remains unknown. However, the CAPL used for the RBC Learn to Play-CAPL project obtained results closely aligned with recent findings from Cycle 2 (2009 to 2011) and Cycle 3 (2012 to 2013) of the Canadian Health Measures Survey (CHMS) [19]. The CHMS is nationally representative and includes physical activity data on children and youth aged 3 to 17 years. Further similarities to our findings are also observed when comparing our results to Cycle 1 (2007 to 2009) of the CHMS [20]. Despite lacking random sampling techniques, we are confident that our results give a reasonable representation of Canadian children’s physical literacy levels.

Additional investigations to evaluate the burden of CAPL-2 for examiners and participants are recommended. Lastly, given that validation is an ongoing process, it is recommended that researchers continue to examine the validity and reliability of scores obtained from CAPL-2, and work to provide criterion-referenced standards to inform revisions to the interpretation of CAPL-2 scores.

## Conclusions

The CAPL-2 offers many benefits for those wishing to assess the physical literacy of children 8 to 12 years of age. Most importantly, it is a comprehensive assessment of all aspects of childhood physical literacy as reflected in the internationally accepted definition. Like the original CAPL, CAPL-2 offers maximum flexibility. Examiners can choose to complete the entire CAPL-2 assessment to provide a comprehensive picture of the child’s physical literacy. However, they can also choose one or more domains, or select individual protocols, if the desire is to examine a particular facet of physical literacy. Regardless of the assessment selected, scores are available to interpret the performance of each child relative to Canadian children of the same age and sex. The CAPL-2 is a streamlined assessment, with a substantially reduced burden for both examiners and participants. The complete CAPL-2 assessment includes three Physical Competence protocols, two Daily Behaviour protocols, and the completion of a 22-response questionnaire. All of the protocols included in the CAPL-2 have published reports of validity and reliability for this age group. The detailed manual for administering the CAPL-2, as well as training materials and other resources, are available free of charge on the CAPL-2 website (www.capl-eclp.ca). All CAPL-2 materials and resources, including the website, are available in both English and French.

## Additional file

Additional file 1: CAPL-2 questionnaire. (PDF 456 kb)

## Abbreviations

CAMSA: Canadian Agility and Movement Skill Assessment
CAPL: Canadian Assessment of Physical Literacy
CAPL-2: Canadian Assessment of Physical Literacy, second edition
CHMS: Canadian Health Measures Survey
GAMLSS: Generalized additive models for location, scale and shape
PA: Physical activity
PACER: Progressive Aerobic Cardiovascular Endurance Run
RBC Learn to Play–CAPL: Royal Bank of Canada Learn to Play–Canadian Assessment of Physical Literacy
