# Supplementary material for: Canadian Assessment of Physical Literacy Second Edition: a streamlined assessment of the capacity for physical activity among children 8 to 12 years of age
Source: BMC Public Health. 2018 Oct 2;18(Suppl 2):1047. doi: 10.1186/s12889-018-5902-y (PMC6167760; doi:10.1186/s12889-018-5902-y)
Supplement: Supplementary file 1 — CAPL-2 questionnaire. (PDF 456 kb) [file 12889_2018_5902_MOESM1_ESM.pdf]

What Do You Think About Physical Activity?

When we ask you about physical activity, we mean when you are moving around, playing or exercising. Physical activity is any activity that makes your heart beat faster or makes you get out of breath some of the time.

Why are we asking you these questions?

We want to know what kids like you think about physical activity, sports and exercise.

Please remember:

- There are no right or wrong answers! We only want to know what you think.
- If you do not know an answer, please write your best guess.
- There is no time limit, so please take all the time you need.

ID: ____________________

What's Most Like Me?

For the first questions you have to read two sentences and then circle the sentence you think is **MORE LIKE YOU**.

Try the following **SAMPLE QUESTION**:

**Some kids have one nose on their face!**	**BUT**	**Other kids have three noses on their face!**

That shouldn't be too hard for you to decide! Once you have circled the sentence that is more like you, then you have to decide if it is **REALLY TRUE** for you or **SORT OF TRUE** for you.

Here is another sample question for you to try. Remember, to answer the question you need to do two things:

1) **First circle the sentence that is more like you.**
2) **Then put a check in the correct box if it is really true or only sort of true for you**.

THERE ARE NO RIGHT OR WRONG ANSWERS, JUST TELL US WHAT YOU THINK IS **MOST LIKE YOU**.

SAMPLE QUESTION #2:

**Some kids like to play with computers**		**BUT**	**Other kids don't like playing with computers**	
**□ REALLY TRUE for me**	**□ SORT OF TRUE for me**		**□ REALLY TRUE for me**	**□ SORT OF TRUE for me**

Now you are ready to start filling in this form**. Remember, in each box you need to circle what is most like you and then check a box for "really" or "sort of" true**. Take your time and do the whole form carefully. If you have any questions, just ask! If you think you are ready you can start now.

BE SURE TO FILL IN EACH PAGE!

What's Most Like Me?

Some kids don't like playing active games		BUT	Other kids really like playing active games	
□ REALLY TRUE for me	□ SORT OF TRUE for me		□ REALLY TRUE for me	□ SORT OF TRUE for me

Some kids are good at active games		BUT	Other kids find active games hard to play	
□ REALLY TRUE for me	□ SORT OF TRUE for me		□ REALLY TRUE for me	□ SORT OF TRUE for me

Some kids don't have much fun playing sports		BUT	Other kids have a good time playing sports	
□ REALLY TRUE for me	□ SORT OF TRUE for me		□ REALLY TRUE for me	□ SORT OF TRUE for me

Some kids do well in most sports		BUT	Other kids feel they aren't good at sports	
□ REALLY TRUE for me	□ SORT OF TRUE for me		□ REALLY TRUE for me	□ SORT OF TRUE for me

Some kids don't like playing sports		BUT	Other kids really enjoy playing sports	
□ REALLY TRUE for me	□ SORT OF TRUE for me		□ REALLY TRUE for me	□ SORT OF TRUE for me

Some kids learn to play active games easily		BUT	Other kids find it hard learning to play active games	
□ REALLY TRUE for me	□ SORT OF TRUE for me		□ REALLY TRUE for me	□ SORT OF TRUE for me

Thank you for telling us which kids are most like you!

We have just a few more questions about physical activity.

Please turn to the next page.

Why are you active?

Boys and girls can be **active** by doing all sorts of things:

- Exercise (walking, keeping fit, or gym class)
- Playing outside or doing active things (like playing in the park)
- Sports (like soccer, tennis, hockey, dance or swimming)

Below are some reasons why you might be active.
Please read each sentence and tell us how true it is for you.

**I am active because...**					
	Not true for me	Not really true for me	Sometimes true for me	Often true for me	Very true for me
being active is fun	€	€	€	€	€
I enjoy being active	€	€	€	€	€
I like being active	€	€	€	€	€

How do you feel about being active?

The next section has some sentences describing how girls and boys feel about BEING ACTIVE and DOING ACTIVE THINGS (like active games, playing outside and doing sports).

Please read each sentence and tell us how much each sentence is like you.

	Not like me at all	Not really like me	Sometimes like me	Quite a lot like me	Really like me
When it comes to playing active games, I think I am pretty good.	€	€	€	€	€
I think I do well at activities compared to other children	€	€	€	€	€
When it comes to being active, I have good skills.	€	€	€	€	€

What do you know about physical activity?

1. How many minutes each day should you and other children do physical activities that make your heart beat faster and make you breathe faster, like walking fast or running? Count the time you should be active at school and also when you are at home or in your neighbourhood.
   a) 20 minutes
   b) 30 minutes
   c) 60 minutes or 1 hour
   d) 120 minutes or 2 hours

2. There are many different kinds of fitness. One type is called endurance fitness or aerobic fitness or cardiorespiratory fitness. Cardiorespiratory fitness means...
   (circle the right answer)
   a) How well the muscles can push, pull or stretch.
   b) How well the heart can pump blood and the lungs can provide oxygen.
   c) Having a healthy weight for our height.
   d) Our ability to do sports that we like.

3. Muscular strength or muscular endurance means...
   (circle the right answer)
   a) How well the muscles can push, pull or stretch.
   b) How well the heart can pump blood and the lungs can provide oxygen.
   c) Having a healthy weight for our height.
   d) Our ability to do sports that we like.

4. If you wanted to GET BETTER AT A SPORT SKILL like kicking and catching a ball, what would be the best thing to do?
   (circle one answer)
   a) Read a book about kicking and catching a ball
   b) Wait until you get older
   c) Try exercising or being active a lot more
   d) Watch a video, take a lesson or have a coach teach you how to kick and catch

5. This story about Sally is missing some words. Choose from the words in the box to fill in the missing words in the story. Each word can only be used to fill one blank space in the story. There are more words than blank spaces, so not all words will be used.

Fun		Stretches	Endurance
Breathing		Flexibility	Good
Pulse	Strength	Bad	Sport

Sally tries to be active every day. Running every day is good for her heart and lungs.

Sally thinks that physical activity is ___________ and is also ___________ for her. At her sport team's practice she does more running to improve her _______________. The team also does exercises like push-ups and sit-ups that increase her _______________. When cooling down, she ______________ to improve her flexibility and slower her heart rate. After exercising, she checks her heart rate which is also called a ____________.

6. During the past week (7 days), on how many days were you physically active for a total of at least 60 minutes per day? (count all of the time you spent doing activities that increased your heart rate or made you breathe hard)

I was active for 0 1 2 3 4 5 6 7 days

Tell us about yourself!

What school grade are you in: (please circle one number)
If you are not in school today, please circle the grade you will be in on the next day that you will go to school.

1 2 3 4 5 6 7 8

Are you a: boy girl (please circle one)

What month is your birthday: (please circle one)

Jan Feb Mar Apr May Jun Jul Aug Sept Oct Nov Dec

How old are you: (please circle one)

5 6 7 8 9 10 11 12 13 14 15

☺ Thank you for answering our questions! ☺
